# High-resolution ultrasound visualization of the recurrent motor branch of the median nerve: normal and first pathological findings

**DOI:** 10.1007/s00330-016-4671-1

**Published:** 2016-12-12

**Authors:** Georg Riegler, Christopher Pivec, Hannes Platzgummer, Doris Lieba-Samal, Peter Brugger, Suren Jengojan, Martin Vierhapper, Gerd Bodner

**Affiliations:** 1 Department of Biomedical Imaging and Image-guided Therapy, Medical University of Vienna, Währingergürtel 18-20, 1090 Vienna, Austria; 2 Department of Neurology, Medical University of Vienna, Währingergürtel 18-20, 1090 Vienna, Austria; 3 Department of Anatomy, Center for Anatomy and Cell Biology, Medical University of Vienna, Währingerstrasse 13, 1090 Vienna, Austria; 4 Department of Surgery, Division of Plastic and Reconstructive Surgery, Medical University of Vienna, Währingergürtel 18-20, 1090 Vienna, Austria

**Keywords:** Median nerve, Carpal tunnel syndrome, Ultrasound, Iatrogenic disease, Anatomical variation

## Abstract

**Purpose:**

To evaluate in a prospective study the possibility of visualization and diagnostic assessment of the recurrent motor branch (RMB) of the median nerve with high-resolution ultrasound (HRUS).

**Materials and methods:**

HRUS with high-frequency probes (18–22 MhZ) was used to locate the RMB in eight fresh cadaveric hands. To verify correct identification, ink-marking and consecutive dissection were performed. Measurement of the RMB maximum transverse-diameter, an evaluation of the origin from the median nerve and its course in relation to the transverse carpal ligament, was performed in both hands of ten healthy volunteers (n = 20). Cases referred for HRUS examinations for suspected RMB lesions were also assessed.

**Results:**

The RMB was clearly visible in all anatomical specimens and all volunteers. Dissection confirmed HRUS findings in all anatomical specimens. Mean RMB diameter in volunteers was 0.7 mm ± 0.1 (range, 0.6–1). The RMB originated from the radial aspect in 11 (55%), central aspect in eight (40%) and ulnar aspect in one (5%) hand. Nineteen (95%) extraligamentous courses and one (5%) subligamentous course were detected. Three patients with visible RMB abnormalities on HRUS were identified.

**Conclusion:**

HRUS is able to reliably visualize the RMB, its variations and pathologies.

***Key Points*:**

• *Ultrasound allows visualization of the recurrent motor branch of the median nerve*.

• *Ultrasound may help clinicians to assess patients with recurrent motor branch pathologies*.

• *Patient management may become more appropriate and targeted therapy could be improved*.

**Electronic supplementary material:**

The online version of this article (doi:10.1007/s00330-016-4671-1) contains supplementary material, which is available to authorized users.

## Introduction

**Fig. 1:**
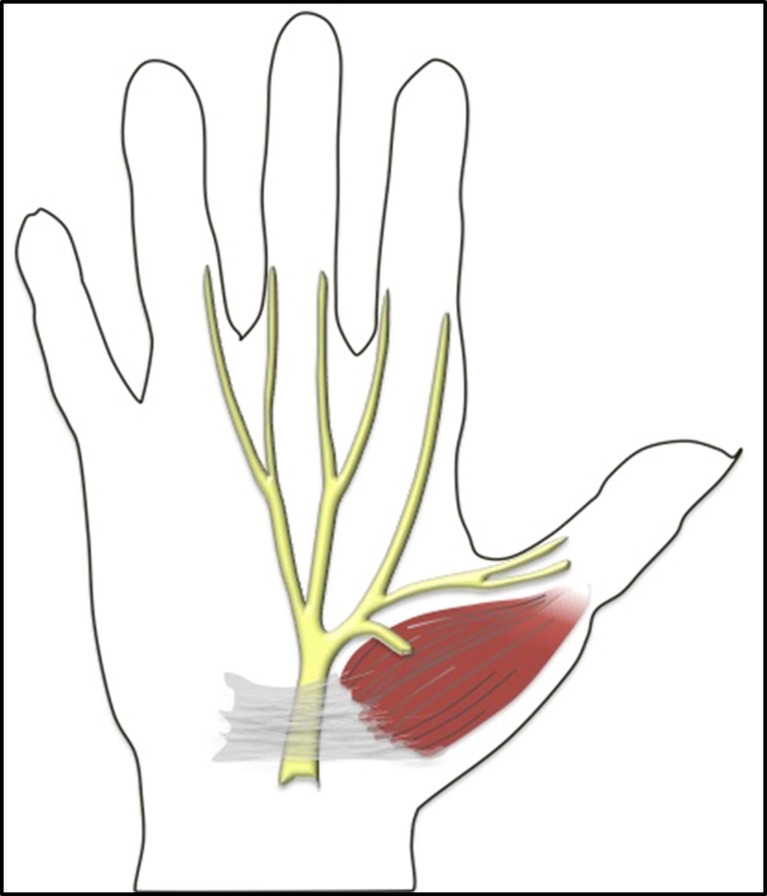
Illustration of the regular branching of the median nerve with an extraligamentous recurrent motor branch coursing toward the thenar musculature

The recurrent motor branch (RMB), sometimes also referred to as the muscular thenar branch of the median nerve, classically supplies innervation to the thenar musculature, including the abductor pollicis brevis, the opponens pollicis and the superficial head of the flexor pollicis brevis (Fig. 1) [1, 2]. These contribute to the most important movements of the hand: opposition and abduction of the thumb.

Damage to the RMB may lead to severely impaired function in patients, with loss of dexterity, pinch and grasp function. The main clinical relevance of the RMB is its susceptibility to iatrogenic injury, due to its variants, during decompression surgery for carpal tunnel syndrome (CTS) [3]. This is because anatomical studies have shown that there is high variability with regard to the origin from the median nerve [4, 5] and its course in relation to the transverse carpal ligament (TCL) [4, 6–9]. Moreover, accessory motor branches have also been described [2, 4, 10–12].

Isolated RMB neuropathies are either rare or possibly underdiagnosed due to the lack of imaging modalities that can depict the nerve. Among these RMB neuropathies, anecdotal reports describe compression of the nerve due to schwannomas [13–15], ganglia [16, 17], anomalous anatomical structures [18, 19], long distance cycling [20] or cutting injuries [21, 22]. Furthermore, there is an ongoing debate about whether selective involvement of the thenar motor fibres is a variant of CTS or an idiopathic entity [23–26].

To date, evaluation and localization of the RMB has been restricted to electrophysiological assessment [26] and clinical testing using landmarks [27, 28].

High-resolution ultrasound (HRUS), using linear, high-frequency probes, offers excellent tissue differentiation for the examination of superficial structures and may facilitate imaging of the RMB. As this has been described for the palmar cutaneous branch of the median nerve [29], which can be assumed to have a comparable diameter, we hypothesized that RMB evaluation would be possible with HRUS. This may open the possibility of diagnosing pathologies related to the nerve, or allow for pre-surgical evaluation or marking in case of suspected variations, and, thus, reduce the risk of iatrogenic injuries.

Therefore, this study aimed to: (i) confirm the correct identification of the RMB by HRUS with ink-marking and consecutive dissection in anatomical specimens; (ii) provide the first measurements of RMB diameter, evaluating the origin, course and possible accessory branches in healthy volunteers; and (iii) present cases with RMB pathology found with HRUS.

## Methods

### Ultrasound technique

This prospective study was approved by the ethics committee of the Medical University of Vienna (EC-number 1529/2015) and was conducted between 1 February 2015 and 1 December 2015.

HRUS examinations were performed using a GE LOGIQ e (GE Healthcare, Wauwatosa, WI, USA) ultrasound (US) platform with high-frequency probes (GE L8-18i-D, GE L10-22-RS). Two radiologists carried out all examinations. One had more than 20 years’ experience (G.B.) and one had 4 years’ experience (G.R.) in peripheral nerve imaging. Both raters were present during the collection of the subjects. G.B. performed all the interventions on all the anatomical specimens. G.R. collected all the images of healthy individuals, with G.R. watching the procedure.

**Fig. 2:**
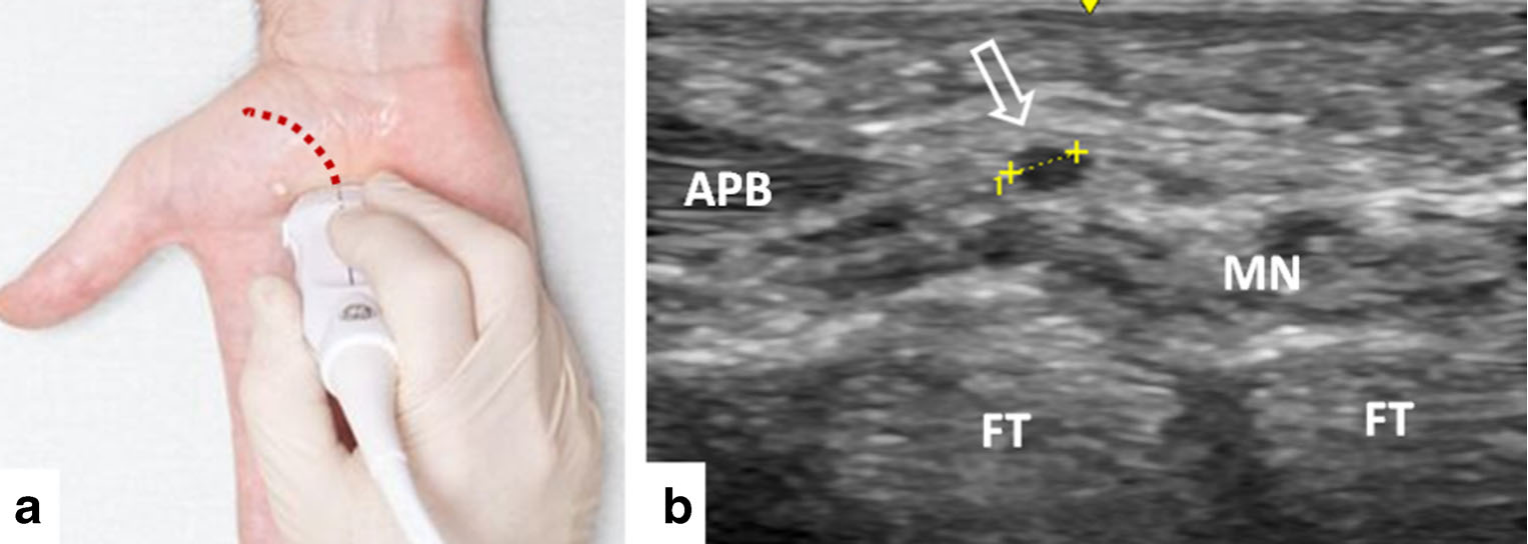
(**a**) Probe positioning at the origin of the recurrent motor branch (RMB). The red dotted line indicates the track of the probe for full RMB examination. To obtain transverse views of the RMB probe, the orientation needs to be perpendicular to the dotted line. (**b**) Example of RMB transverse diameter measurement (arrow) in a healthy volunteer (0.8 mm). *APB* abductor pollicis brevis muscle, *FT* flexor tendon, *MN* median nerve

**Fig. 3:**
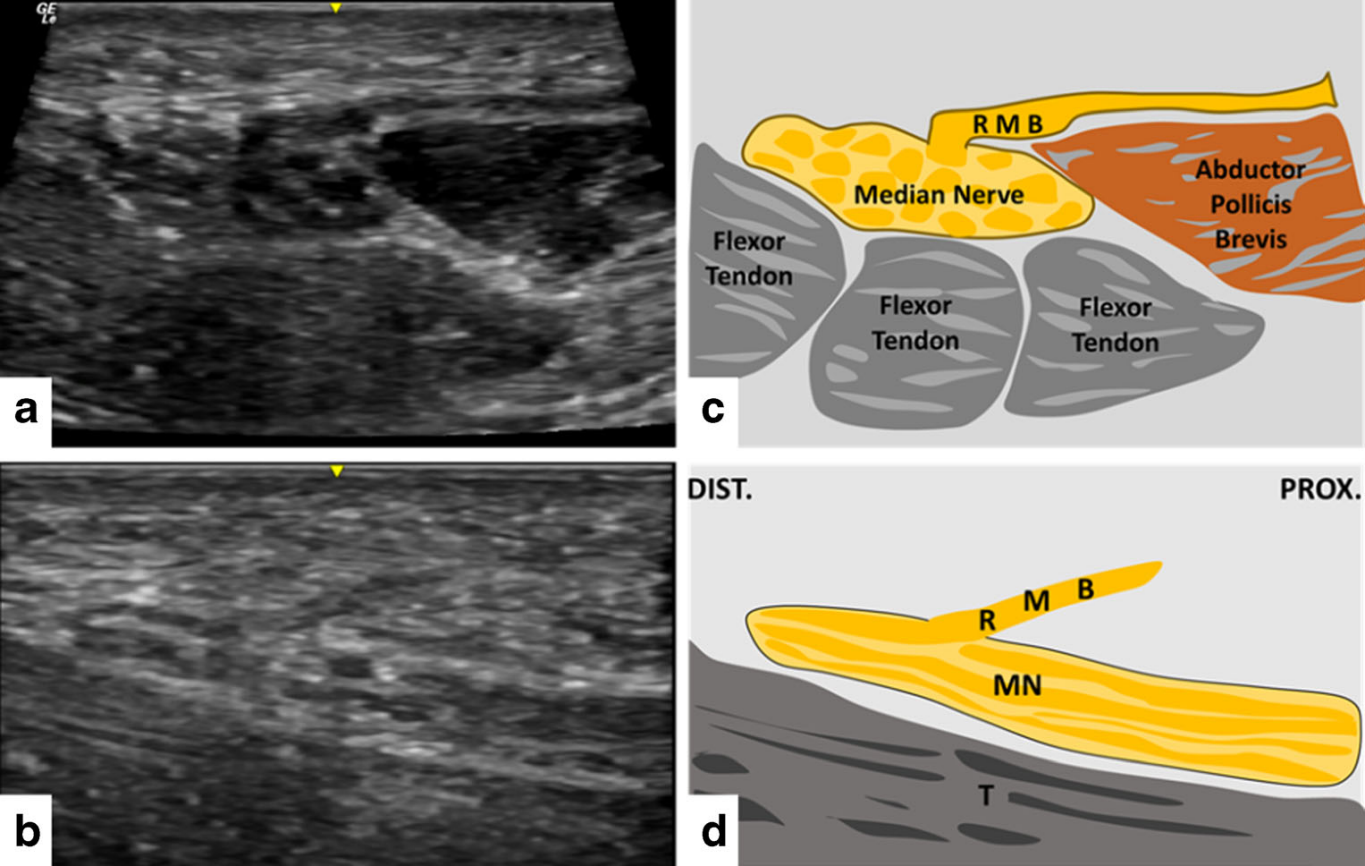
(**a**, **b**) Example of sonographic findings of the recurrent motor branch (RMB) at its origin from the central aspect of the median nerve in a radio-ulnar transverse view. (**c**, **d**) Example of sonographic findings on a long-axis view of the RMB at its origin from the central aspect of the median nerve coursing proximally toward the thenar. The median nerve and flexor tendons are obliquely projected. Note the approximately 45° angle of the RMB with the median nerve. *FT* flexor tendon, *MN* median nerve, *DIST*. distal, *PROX*. proximal

The examination followed a standardized assessment protocol that started with the transverse view of the median nerve or its digital cutaneous branches at the level of the metacarpal bodies III-IV. The probe was moved proximally until a tubular structure arising from the median nerve, in most cases curving radially and proximally and coursing toward the thenar musculature, was presumed to be the RMB. Subsequently, the origin was assessed by turning the probe until the longitudinal axis of the nerve was visible. In most cases, the RMB formed an approximately 45° angle with the median nerve. Subsequently, the nerve was followed until its entrance into the thenar musculature. To avoid confusion with the palmar cutaneous branch or the palmar digital branch of the median nerve, the RMB had to enter the thenar musculature in contrast to the other branches. To avoid confusion with vessels, colour Doppler was used. Probe positioning, probe track and measurement of the RMB diameter is presented in Fig. 2. The normal presentation of the RMB at its origin is presented in Fig. 3.

A second possibility to locate the RMB (considered by the authors to be more difficult) was to start with the transverse view of the median nerve 3 cm proximal to the pisiform bone. Following the median nerve, the probe was moved distally until its subdivision into terminal branches. At this level, the probe was moved proximally and distally to identify the RMB. After identification, the assessment of the nerve was performed in a manner similar to that described above.

### Ultrasound in anatomical specimens

In four randomly selected fresh anatomical specimens in the legal custody of the Department of Systematic Anatomy, Medical University of Vienna, HRUS was performed as described above in both wrists (n = 8). After locating the RMB, a small amount of blue dye mixed with glue (0.1 ml) was injected into the nerve/adjacent to the nerve under HRUS guidance (22-gauge needle, in-plane technique). Subsequent anatomical dissection was performed to confirm the exact location of the dye injection. A plastic surgeon (M.V.) and anatomist (P.B.) who performed the dissections determined the exact location of the dye injection. Correct dye injection was noted if at least some amount of the dye was injected into the nerve sheath.

### Ultrasound in healthy volunteers

Ten healthy volunteers were recruited via notices at the Department of Biomedical Imaging and Image-guided Therapy and word-of-mouth acquisition. Written informed consent was obtained from all volunteers. Inclusion criteria were age over 18 years, and exclusion criteria were known polyneuropathy, known myopathy, chronic disease known to cause peripheral neuropathy, current or previous CTS and previous hand surgery.

The RMB was assessed on both sides (n = 20). Measurements of the maximum transverse diameter were obtained immediately after the separation from the median nerve using the platform software of LOGIQ e. The origin of the RMB with respect to the median nerve was assessed according to Mackinnon and Dellon (4). The site of origin of the RMB from the median nerve was classified as either ulnar, central-volar or radial, since the intermediate type (one-third the distance between the radial and central aspect) described by Mackinnon and Dellon [5] is not clearly distinguishable with US.

The course of the RMB in relation to the transverse carpal ligament (TCL) was evaluated using the classification of Lanz [8]: extraligamentous – origin and course distal to the TCL; subligamentous – origin within the carpal tunnel, winding around the distal edge of the TCL; transligamentous – piercing the TCL. Furthermore, the presence of accessory branches was evaluated.

### Ultrasound in patients

Between 1 February 2015 and 1 December 2015, we monitored patients who were referred to the Department of Biomedical Imaging and Image-guided Therapy, and in whom RMB pathologies were detected with HRUS. The referring diagnosis of all screened patients was clinically or electrophysiologically diagnosed carpal tunnel syndrome. All patients were referred to our department for preoperative evaluation prior to carpal tunnel surgery. HRUS examinations were performed using the same assessment protocol as described above.

### Statistical analysis

Descriptive statistics were performed using IBM SPSS Statistics for Windows Version 22.0.0.2 (IBM, Armonk, NY, USA). Metric data (nerve diameter) are presented as mean ± standard deviation and range (minimum–maximum).

## Results

### Ultrasound in anatomical specimens

The RMB was clearly visible in all anatomical specimens. Dissection confirmed the correct identification of the RMB (100%) on both sites in all subjects (n = 8). An example of a dissection finding is shown in Fig. 3.

### Ultrasound in healthy volunteers

Table 1 shows a summary of all demographic findings and measurements. Five females and five males (mean age, 31.5 years; age range, 27–54 years) were included in the study. The RMB could be visualized in both wrists of all volunteers (n = 20). Assessment of the nerve was possible from its origin until its ramification into terminal branches. Some of these branches could even be visualized in the thenar musculature. Sonographically, the RMB appeared as a hypoechoic, round dot in the transverse view, with a small surrounding hyperechoic border. While the hypoechoic dot was clearly depictable, the surrounding hyperechoic tissue, which we presumed to be the epineurium, was not well distinguishable from the adjacent tissue. Individual fascicles of the RMB could be seen in only a few cases.

**Table 1:** Demographic characteristics, measurement of transverse diameter, origin, course, and branches of the RMB in healthy

Volunteer No.	Sex	Age (y)	Side	TD (mm)	Origin	Branches	Course
1	M	31	R	0.06	Cen	1	Extra
1	M	31	L	0.07	Rad	1	Extra
2	M	33	R	0.06	Rad	1	Extra
2	M	33	L	0.06	Rad	1	Extra
3	M	55	L	0.07/0.06(a)	Rad	2	Extra
3	M	55	R	0.06	Rad	1	Extra
4	F	38	R	0.10	Rad	1	Extra
4	F	38	L	0.07	Uln	1	Sub
5	F	31	R	0.07	Cen	1	Extra
5	F	31	L	0.08	Cen	1	Extra
6	F	30	R	0.07	Rad	1	Extra
6	F	30	L	0.06	Cen	1	Extra
7	F	54	R	0.07	Cen	1	Extra
7	F	54	L	0.07	Cen	1	Extra
8	M	32	R	0.10	Rad	1	Extra
8	M	32	L	0.07	Rad	1	Extra
9	F	27	R	0.07	Rad	1	Extra
9	F	27	L	0.07	Cen	1	Extra
10	M	31	R	0.10	Rad	1	Extra
10	M	31	L	0.07	Cen	1	Extra

*RMB* recurrent motor branch, *No*. number, *M* male, *F* female, *y* years, *R* right, *L* Left, *mm* miliimeter, *TD* transverse diameter/mean cross sectional diameter, (*a*) accessory branch, *Cen* central, *Rad* radial, *Uln* ulnar, *Extra* extraligamentous, *Sub* subligamentous

The mean transverse diameter was 0.7 mm ± 0.1 (range, 0.6–1). The maximum detectable intraindividual side difference was 0.03 mm.

The RMB originated from the radial aspect in 11 hands (55%), the central aspect in eight hands (40%), and the ulnar aspect in one hand (5%).

**Fig. 4:**
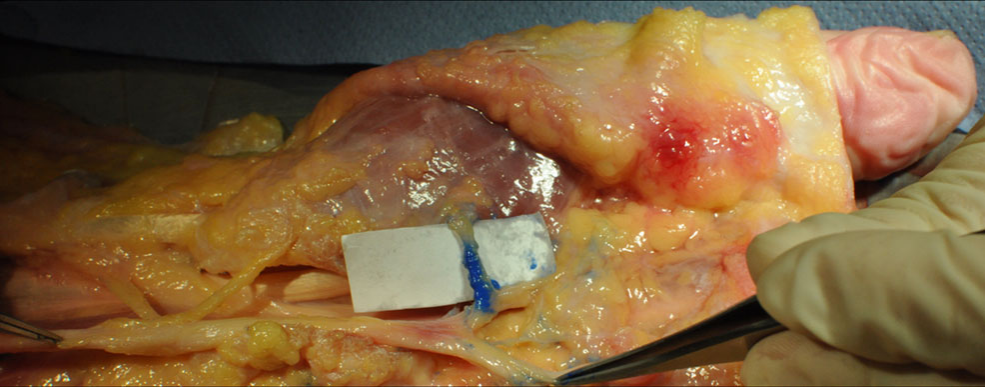
Example of finding in a dissection after high-resolution ultrasound (HRUS)-guided, intraneural ink-marking of the recurrent motor branch

An extraligamentous course was seen in 19 hands (95%), and a subligamentous course in one hand (5%). No transligamentous course was observed. One accessory branch arising from the radial aspect of the median nerve, with a maximum transverse diameter of 0.6 mm, was detected (Fig. 4 and Movie).

### Ultrasound in patients

**Fig. 5:**
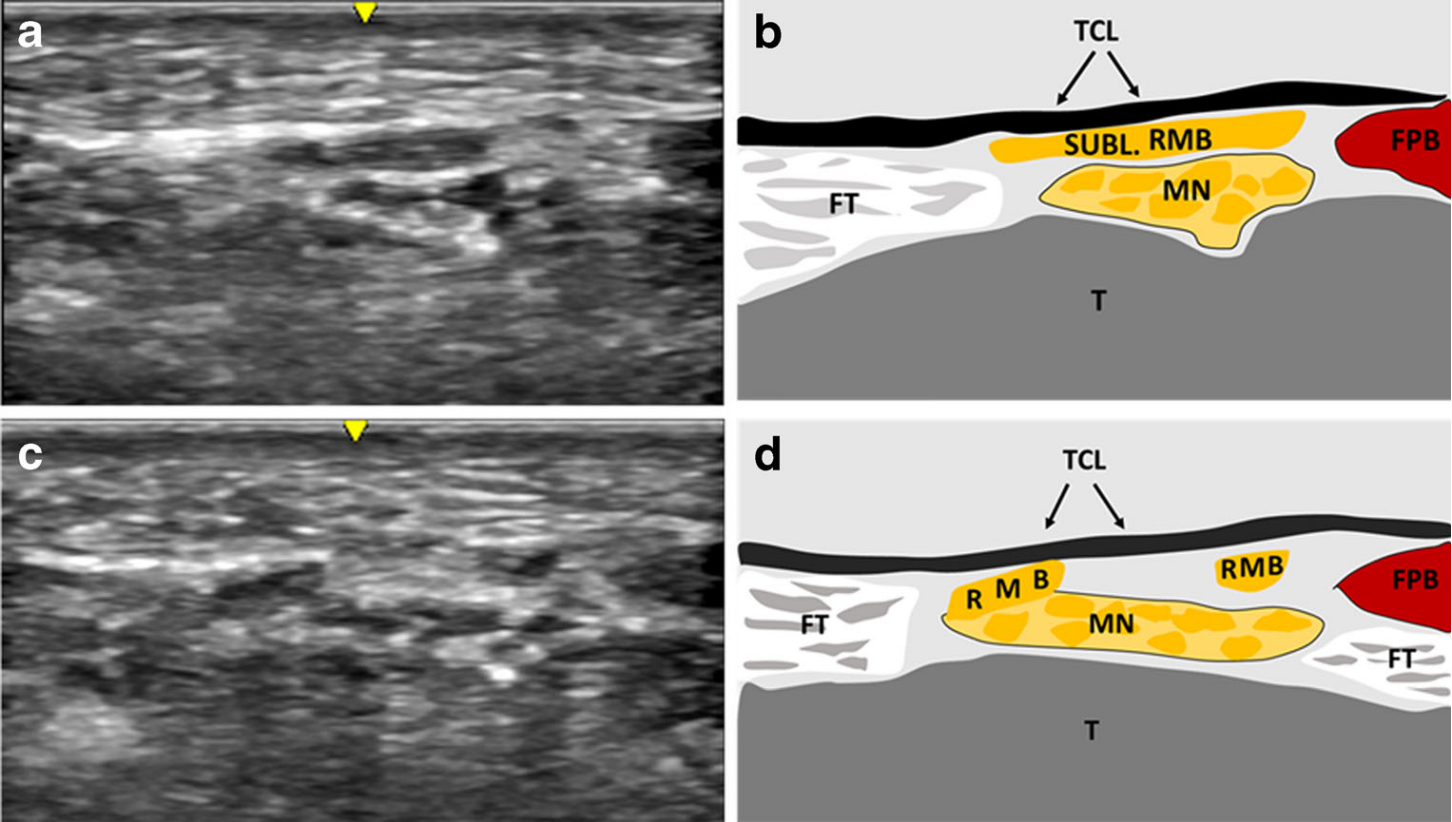
(**a**, **b**) Example of sonographic findings of the recurrent motor branch (RMB) originating from the ulnar aspect of the median nerve. (**c**, **d**) Example of sonographic findings in the same volunteer. The RMB crosses the anterior aspect of the median and courses beneath the transverse carpal ligament (TCL; subligamentous) toward the thenar musculature. *FPB* flexor pollicis brevis muscle, *FT* flexor tendon, *MN* median nerve, *SUBL* subligamentous

Of 189 patients with carpal tunnel syndrome, three patients (1.6%) with RMB pathologies were identified and are presented below. Figure 5 shows the HRUS findings of all patients.

#### Case 1

A 51-year-old female presented with a weakening of the thenar musculature for the 6 months prior to presentation, at the left wrist, sometimes combined with slight pain and paraesthesias in the first finger on her left side. Clinical examination revealed a loss of power (0/5) of thumb abduction, thenar wasting and a mild hypoesthesia of the first digit. Motor conduction studies showed severe axonal damage of motor fibres on the left (compound muscle action potential: left 1,500 μV, right 18,700 μV) and prolongation of distal motor latency, while sensory testing revealed a preserved sensory nerve action potential with a only slightly reduced amplitude of 12 μV and a decreased antidromic conduction velocity of 44 m/s. Sonographic assessment revealed a radially originating, extraligamentously coursing and severely thickened RMB (1.5 mm vs. 0.9 mm on the right). Further, the whole median nerve also exhibited an increased cross-sectional area of 0.23 cm^2^ within/distal to the carpal tunnel (upper limit normal ≤0.12 cm^2^). The clinically visible atrophy was also documented with US (Fig. 5). In accordance with the present findings, this was assumed to be CTS with severe involvement of the RMB.

#### Case 2

A 49-year-old female presented with paresis of the thenar muscles on her right side for the 4 months prior to presentation. No paraesthesias or pain were reported. Clinical examination revealed paresis of thumb opposition (2/5) and mild paresis of thumb abduction (4/5). Atrophy of the lateral thenar and the opponens pollicis muscle was visible. Sonographic assessment revealed a radially originating, extraligamentously coursing, moderately thickened RMB (1.3 mm) and a normal cross-sectional area of 0.10 cm^2^ (standard value ≤0.12 cm^2^) of the median nerve. Further, atrophy of the thenar musculature was assessed (Fig. 5). The patient was operated upon approximately 2 months after HRUS examination. There was a positive correlation between the sonographic findings and surgery. Intraoperatively, the RMB was thickened and seemed to be entrapped in ‘fibrous tissue’ directly after its origin from the median nerve, as classified by the surgeons. The fibrous tissue was removed and neurolysis was performed. A short-term follow-up 5 weeks after surgery revealed improved power (3/5) of thumb opposition and thumb abduction (5/5). In accordance with the present findings, this was assumed to be a variant of CTS with involvement of the RMB.

#### Case 3

**Fig. 6:**
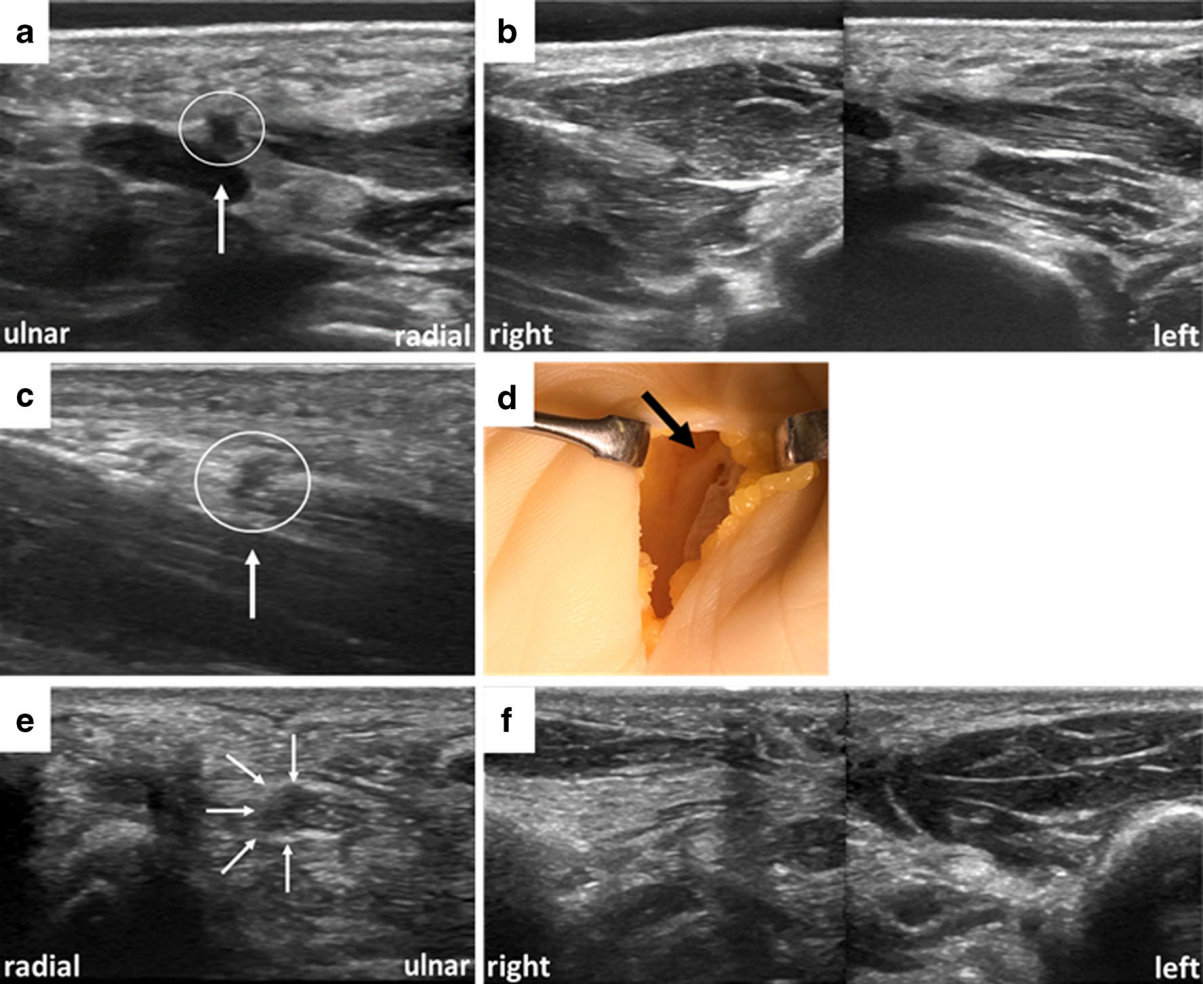
(**a**, **b**) Findings in patient 1, showing an enlarged recurrent motor branch (RMB) (encircled) atop the enlarged median nerve just distal to the carpal tunnel and a clear thenar atrophy on the patient’s left side. (**c**, **d**) Findings in patient 2, showing a thickened RMB (encircled). The median nerve is obliquely projected. (**d**) Intraoperative findings of the RMB after removal of fibrous tissue surrounding the branch. (**e**, **f**) Findings in patient 3, with swollen radial-sided motor fascicles within the median nerve and a clear thenar atrophy on the patient’s right side

A 45-year-old female presented with a severe paresis of the thenar musculature on her right side for the 6 months prior to presentation. No paraesthesias or pain were reported. Clinical examination revealed plegia of thumb abduction (0/5). A clear thenar atrophy was visible. Sonographic assessment revealed a radially originating, extraligamentously coursing and moderately thickened RMB (1.3 mm). Moreover, the radial-sided motor fascicles within the median nerve were clearly swollen. Further, atrophy of the thenar muscles was assessed (Figs. 5 and 6). In accordance with the present findings, this was assumed to be a variant of CTS with involvement of the RMB.

## Discussion

This study confirms the reliable visualization of the RMB with HRUS using US-guided ink-marking and consecutive dissection in a series of anatomical specimens. Initial measurements of the RMB in healthy volunteers showed a mean transverse diameter of 0.7 mm ± 0.1 mm (range, 0.6–1). Subsequently, a broad variation in point of origin and course was observed.

Ultrasound measurements of the transverse diameter of the RMB in healthy volunteers revealed clearly lower values than previously described in anatomical studies. Wang and colleagues [30] reported a mean diameter of 1.7 ± 0.3 mm in seven fresh forearm amputation specimens, and Üstün et al. [31] reported a mean diameter of 1.4 mm ± 0.12 mm in ten fresh cadaver arms. This discrepancy in nerve diameter may be due to the different visualization methods employed. In our study, we could measure only the hypoechoic pattern of the nerve without the surrounding neural tissue. Measurements of the transverse diameter of the RMB with US have not yet been reported. As presented, the transverse diameter in patients clearly exceeded the upper limit observed in healthy volunteers. Therefore, HRUS may help to evaluate RMB pathologies, and our data may serve as a reference for further, more detailed US characterizations of the RMB.

In addition to the depiction of the RMB, HRUS provides further information about the course, origin and branching of this nerve. The course of the RMB in relation to the TCL has gained extensive attention in various studies, due to the fact that the ‘anomalous’ trans- and subligamentous variants, in particular, are at risk during both open and endoscopic surgery [3, 7, 8, 32]. Our results are comparable with previous studies [4, 6–9] (extraligamentous, 46–97%; subligamentous, 2–34%; transligamentous, 1–23%), although we did not detect a transligamentous course, which is mainly attributable to the small study sample.

The origin from the radial aspect of the median nerve was described as a possible site of entrapment because of separate obliquely arranged fibres from the TCL encircling the RMB [7]. As the radial origin is most common and the results of previous studies are in accordance with our data (55% vs. 60–80%) [4, 5], we think this condition can be easily detected by HRUS. The less frequent origin from the ulnar aspect of the median nerve (5% in our study vs. 1.1% in a study of 821 hands that had undergone carpal tunnel (CT) release surgery) [9] represents a major risk for iatrogenic injury with the ulnar side approach, as well as with the median approach, since the nerve crosses the anterior aspect of the median nerve during CT release [33].

Iatrogenic RMB injury during CT release seems to be a rare complication, with approximately 0.5% in the reported literature [3, 32]. Nevertheless, it represents a severe complication also called the ‘million dollar injury’ due to the compensation awarded in lawsuits because of the loss of thenar function [34, 35]. To date, no consensus exists about whether the branch should be examined intra- or preoperatively to avoid damage. Of 153 surgeons responding to a questionnaire, the majority (>70%) did not explore the nerve routinely and did not recommend doing so [36]. Other authors suggest exploring the RMB intraoperatively, at least for some special conditions [37]. Indeed, whenever the surgeon encounters muscle fibres lying superficial to or interposed within the TCL, there is a greater than 90% likelihood that the motor branch would be anomalous [37]. The preoperative RMB localization is limited to the use of surface landmarks, such as the Kaplan’s cardinal and middle finger radial-side-line, or a physical examination manoeuvre, such as the middle finger flexion test [27, 28]. Nevertheless, these tests are inaccurate when the RMB has a varying course. As an example, in middle finger flexion tests, the transligamentous course showed a deviation of 10–25 mm from where it was expected [27, 28]. RMB evaluation with HRUS overcomes all these limitations. It allows visualization of the nerve along its entire course, which may help surgeons to plan their approach for CT release. In the case of a ‘dangerous’ variation, preoperative skin-marking could be provided to facilitate exploration of the nerve. Although we did not observe an iatrogenic injury prior to re-operation during our short observation period, we saw one patient postoperatively who was treated for a complete transection of the RMB during carpal tunnel release. Therefore, the results of this study suggest that HRUS evaluation of the RMB should be included as part of the conventional sonographic examination for CTS to minimize iatrogenic injury during CT release.

In 1982, Bennet and Crouch [23] reported two cases of isolated compression of the RMB, characterized by selective involvement of thenar motor fibres. In these cases, the surgical observation showed compression of the branch due to a transligamentous course or an excessive angle of the thenar branch at the distal edge of the transverse ligament with neuroma formation proximal to the entrapment sites. Four subsequent electrophysiological and clinical studies underlined the theory that motor fascicles alone may be involved in CTS, or represent a separate entity without the classic CTS [24–26, 38]. Our case 2 and case 3 may provide a hint that these conditions may be detectable by HRUS in the future. Nevertheless, further comparative HRUS studies between normal and pathological RMB conditions are needed to reliably answer this question.

This study has several strengths and limitations. Its strengths include the first-time use of HRUS for specific assessment of the RMB and confirmation of the findings by the gold standard of anatomical dissection. Its limitations include the fact that findings *in vivo* were uncontrolled. However, accuracy in anatomical specimens was 100% and RMB in volunteers could be followed into the abductor muscle. A further limitation is the fact that pathological findings in this manuscript are case reports and do not provide reliable information about the future role of HRUS in RMB pathology detection. For this reason, further comparative studies between normal and pathological conditions are needed.

In conclusion, this study confirms the reliable ability to visualize the RMB and its variations with HRUS, in anatomical specimens and in healthy volunteers. We therefore encourage the use of HRUS, especially for preoperative evaluation for carpal tunnel release or if thenar muscle weakening is present. Further studies are needed to assess the value of HRUS in diagnosing RMB pathologies.

## Electronic supplementary material

Below is the link to the electronic supplementary material.

Movie Anatomical variant. Video sequence of sonographic findings in a volunteer with a recurrent motor branch originating from the ulnar aspect of the median nerve, crossing the anterior aspect of the median and coursing beneath the TCL (subligamentous) toward the thenar musculature (MP4 40229 kb)
